# Supplementary figures and images for: The illicit cigarette market in the Democratic Republic of the Congo (DRC): Findings from a cross-sectional study of empty cigarette packs
Source: PLOS Glob Public Health. 2025 Jun 25;5(6):e0003937. doi: 10.1371/journal.pgph.0003937 (PMC12194187; doi:10.1371/journal.pgph.0003937)

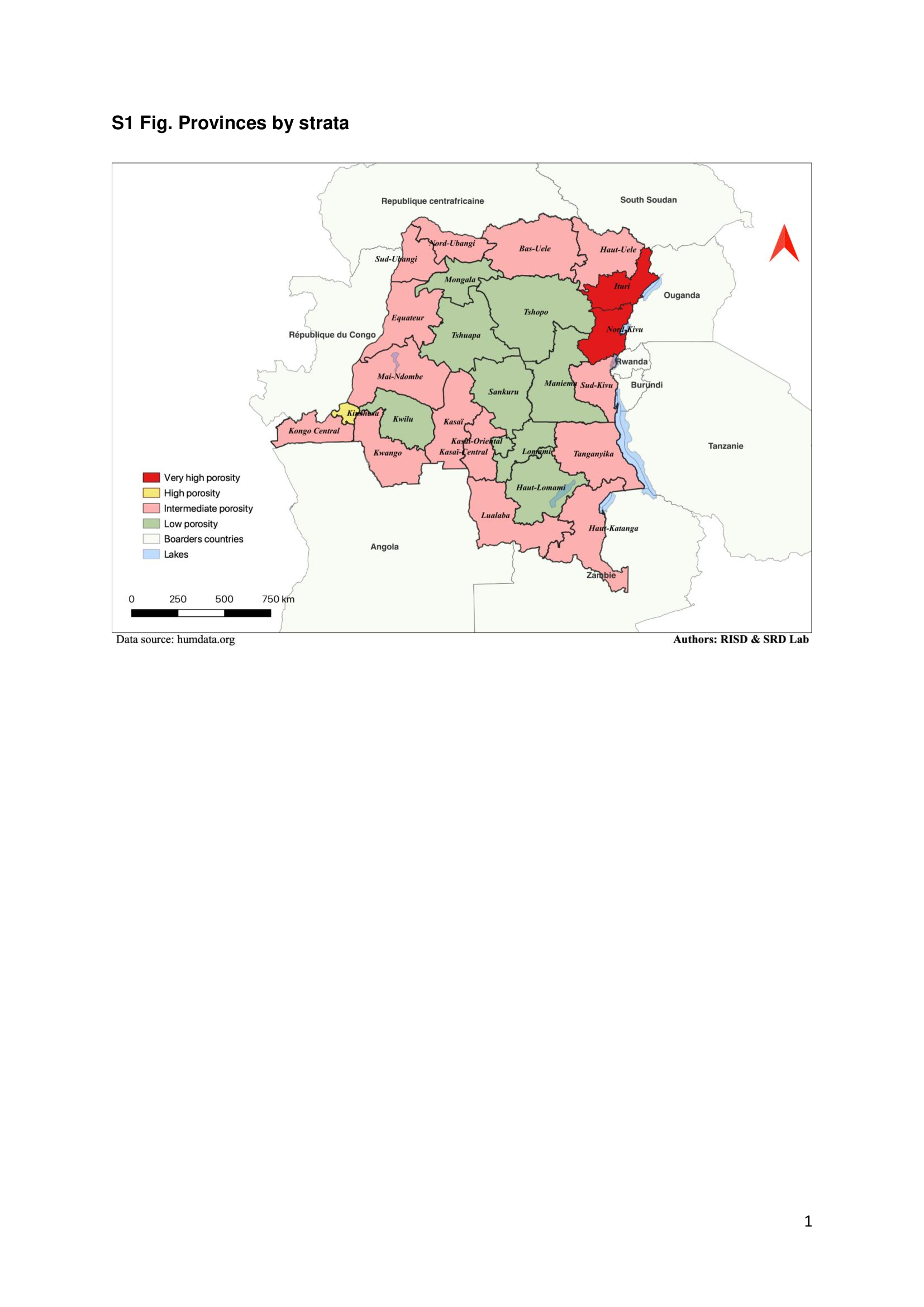

Supplement: S1 Fig — (TIFF) [file pgph.0003937.s001.tiff]

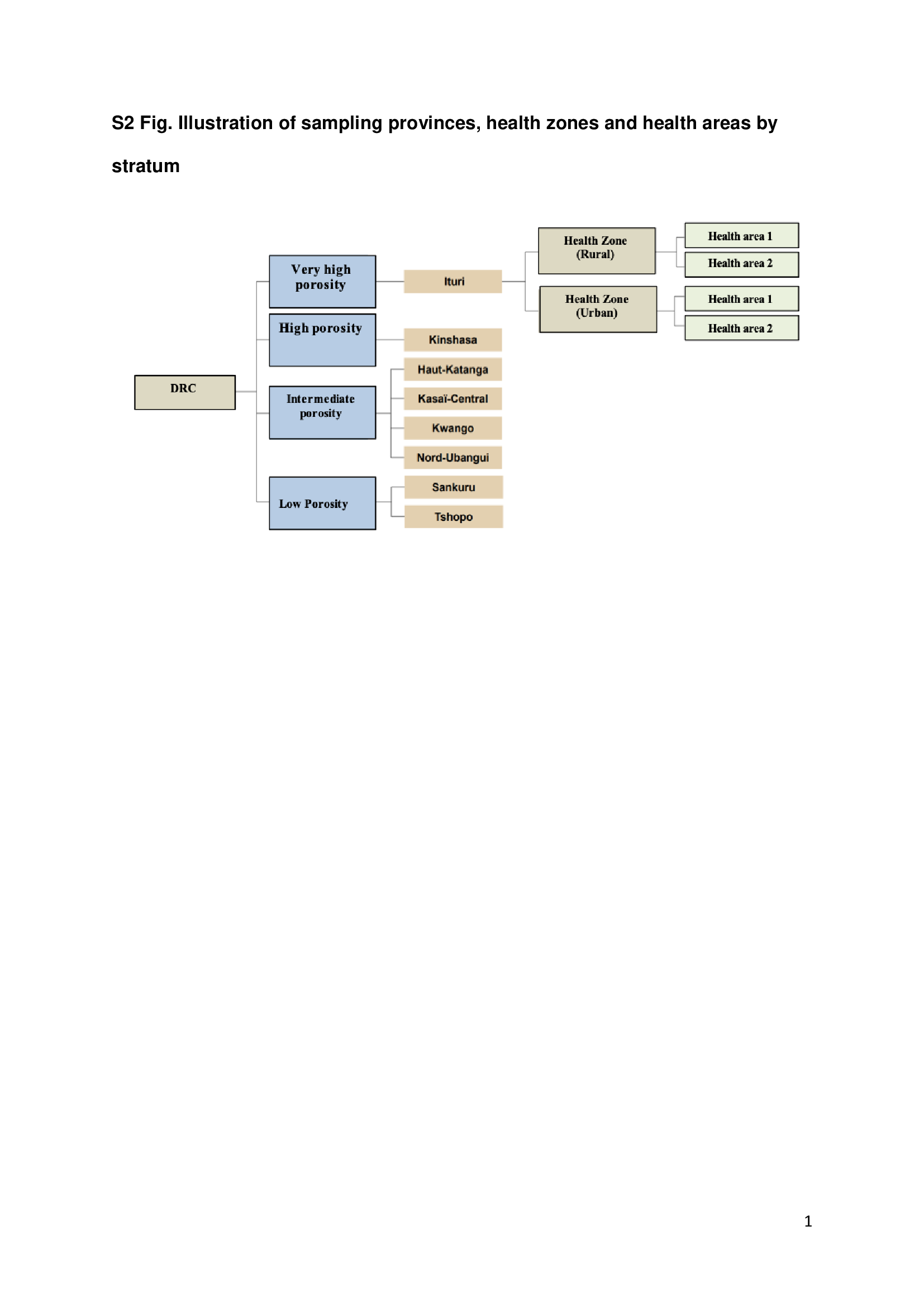

Supplement: S2 Fig — (TIFF) [file pgph.0003937.s002.tiff]
