# Supplementary material for: The illicit cigarette market in the Democratic Republic of the Congo (DRC): Findings from a cross-sectional study of empty cigarette packs
Source: PLOS Glob Public Health. 2025 Jun 25;5(6):e0003937. doi: 10.1371/journal.pgph.0003937 (PMC12194187; doi:10.1371/journal.pgph.0003937)
Supplement: S1 Text — (DOCX) [file pgph.0003937.s003.docx]

**S1 Table. Participating health zones and health areas by province and urban/rural classification**

| **Province** | **Urban/rural** | **Health Zone** | **Health Area** |
| --- | --- | --- | --- |
| Haut Katanga | Rural | Kambove | Dikula |
|  |  |  | Kiwewe |
|  | Urban | Tshamilemba | Cimenkat |
|  |  |  | Rail |
| Kinshasa | Urban | Binza Ozone | Munganga |
|  |  |  | Mama Yemo |
|  | Rural | Mount Ngafula 2 | Kimbondo |
|  |  |  | Antenne |
| Tshopo | Urban | Mangobo | Tp Maman Mwilu |
|  |  |  | Tp Profession |
|  | Rural | Banalia | Mosanda |
|  |  |  | Babise |
| Sankuru | Rural | Minga | Engo Dihoka |
|  |  |  | Ohale |
|  | Urban | Lusambo | Lusambo Ouest |
|  |  |  | Lusambo Est |
| Kwango | Urban | Zs Kenge | Saint Esprit |
|  |  |  | Cbco |
|  | Rural | Zs Kahemba | Bumba |
|  |  |  | Tshifwameso |
| Kasaï-Central | Urban | Bobozo | District Est |
|  |  |  | Kamayi |
|  | Rural | Bilomba | Tshikupela |
|  |  |  | Kabawu |
| Ituri | Urban | Bunia | Mudzi Maria |
|  |  |  | Bunia Cité |
|  | Rural | Rethy ^*^ | Gudjo |
|  |  |  | Mbr'bu |
| Nord Ubangui | Urban | Gbadolite | Bolingo |
|  |  |  | Gbadolite |
|  | Rural | Bosobolo | Kwala |
|  |  |  | Bok.Pombo |
