# Supplementary material for: The illicit cigarette market in the Democratic Republic of the Congo (DRC): Findings from a cross-sectional study of empty cigarette packs
Source: PLOS Glob Public Health. 2025 Jun 25;5(6):e0003937. doi: 10.1371/journal.pgph.0003937 (PMC12194187; doi:10.1371/journal.pgph.0003937)
Supplement: S2 Text — (DOCX) [file pgph.0003937.s004.docx]

**S2 Table. Proportion of illicit empty packs by type of collection point, porosity and province with all imported yellow stamps as illicit**

|  | **Collection points** | | | | | | | | | | | |
| --- | --- | --- | --- | --- | --- | --- | --- | --- | --- | --- | --- | --- |
| **Porosity**  Province | **Stationary retailer** | | **Mobile retailer** | | **Garbage bins/streets** | | | | **Total** | | | |
|  | Illicit  n  % (95% CI) | Total | Illicit  n  % (95% CI) | Total | Illicit  n  % (95% CI) | Total | | | Illicit  n  % (95% CI) | Total | | |
| **Very high** |  |  |  |  |  | | |  |  | |  | |
| Ituri | 1,057 | 1,333 | 21 | 29 | 102 | | | 117 | 1,180 | | | 1,479 |
|  | 79.3 (77.0, 81.4) |  | 72.4 (52.8, 87.3) |  | 87.2 (79.7, 92.6) | | |  | 79.8 (77.6, 81.8) | | |  |
| **High** |  |  |  |  |  | | |  |  | | |  |
| Kinshasa | 656 | 918 | 383 | 524 | 218 | | | 298 | 1,257 | | | 1,740 |
|  | 71.5 (68.4, 74.4) |  | 73.1 (69.1, 76.8) |  | 73.2 (67.7, 78.1) | | |  | 72.2 (70.1, 74.3) | | |  |
| **Intermediate** | **1,612**  **43.5 (41.9, 45.2)** | **3,702** | **414**  **44.0 (40.8, 47.2)** | **941** | **686**  **64.8 (61.8, 67.6)** | | | **1,059** | **2,712**  **47.6 (46.3, 48.9)** | | | **5,702** |
| Haut-Katanga | 278 | 424 | 43 | 129 | 217 | | | 361 | 538 | | | 914 |
|  | 65.5 (60.8, 70.1) |  | 33.3 (25.3, 42.2) |  | 60.1 (54.9, 65.2) | | |  | 58.9 (55.6, 62.1) | | |  |
| Kasai-central | 380 | 878 | 133 | 326 | 76 | | | 183 | 589 | | | 1,387 |
|  | 43.3 (40.0, 46.6) |  | 40.8 (35.4, 46.3) |  | 41.5 (34.3, 49.0) | | |  | 42.5 (39.8, 45.1) | | |  |
| Kwango | 582 | 879 | 238 | 486 | 375 | | | 448 | 1,195 | | | 1,813 |
|  | 66.2 (63.0, 69.3) |  | 49.0 (44.4, 53.5) |  | 83.7 (80.0, 87.0) | | |  | 65.9 (63.7, 68.1) | | |  |
| Nord-Ubangi | 372 | 1,521 | 0 | 0 | 18 | | | 67 | 390 | | | 1,588 |
|  | 24.5 (22.3, 26.7) |  | // (//) |  | 26.9 (16.8, 39.1) | | |  | 24.6 (22.5, 26.8) | | |  |
| **Low** | **217**  **18.7 (15.3, 22.3)** | **1,163** | **5**  **17.9 (6.1, 36.9)** | **28** | **95**  **18.6 (15.3, 22.3)** | | | **510** | **317**  **18.6 (16.8, 20.6)** | | | **1,701** |
| Sankuru | 145 | 387 | 5 | 10 | 70 | | | 186 | 220 | | | 583 |
|  | 37.5 (32.6, 42.5) |  | 50.0 (18.7, 81.3) |  | 37.6 (30.7, 45.0) | | |  | 37.7 (33.8, 41.8) | | |  |
| Tshopo | 72 | 776 | 0 | 18 | 25 | | | 324 | 97 | | | 1,118 |
|  | 9.3 (7.3, 11.5) |  | 0.0 (0.0, 19.0) |  | 7.7 (5.1, 11.2) | | |  | 8.7 (7.1, 10.5) | | |  |
| **Total** | **3,542** | **7,116** | **823** | **1,522** | **1,101** | | **1,984** | | **5,466** | **10,622** | | |
|  | **49.8 (48.6, 50.9)** |  | **54.1 (51.5, 56.6)** |  | **55.5 (53.3, 57.7)** | |  | | **51.5 (50.5, 52.4)** | | |  |
