# Supplementary material for: The illicit cigarette market in the Democratic Republic of the Congo (DRC): Findings from a cross-sectional study of empty cigarette packs
Source: PLOS Glob Public Health. 2025 Jun 25;5(6):e0003937. doi: 10.1371/journal.pgph.0003937 (PMC12194187; doi:10.1371/journal.pgph.0003937)
Supplement: S3 Text — (DOCX) [file pgph.0003937.s005.docx]

**S3 Table. Proportion of illicit empty packs by rural/urban, area-level income group, brand and country of origin with all imported yellow stamps as illicit**

|  | **Total packs collected** | **Number of illicit packs** | **Proportion of illicit packs** |
| --- | --- | --- | --- |
| **Urban/rural** |  |  |  |
| Rural | 2,704 | 1,296 | **47.9** |
| Urban | 7,918 | 4,170 | **52.7** |
| **Area-level income group** |  |  |  |
| High-income | 3,492 | 2,052 | **58.8** |
| Low-income | 7,130 | 3,414 | **47.9** |
| **Brand** |  |  |  |
| Supermatch | 894 | 504 | **56.4** |
| Oris | 532 | 439 | **82.5** |
| Pall Mall | 1,238 | 1,188 | **96.0** |
| Monte Carlo | 1,914 | 1,913 | **100.0** |
| Stella | 546 | 541 | **99.1** |
| Business | 147 | 147 | **100.0** |
| Caesar | 120 | 4 | **3.3** |
| Ambassade | 240 | 240 | **100.0** |
| Elite | 525 | 0 | **0.0** |
| Equateur | 2,109 | 0 | **0.0** |
| Master | 1,809 | 0 | **0.0** |
| Portsman | 262 | 257 | **0.0** |
| Other brands | 286 | 233 | **81.5** |
| **Country of origin** |  |  |  |
| DRC | 4,834 | 0 | **0.0** |
| Uganda | 403 | 403 | **100.0** |
| United Arab Emirates | 743 | 641 | **86.2** |
| South Sudan | 64 | 64 | **100.0** |
| Kenya | 2,108 | 2,052 | **97.3** |
| India | 16 | 16 | **100.0** |
| Tanzania | 2,178 | 2,173 | **99.8** |
| South Africa | 162 | 5 | **3.1** |
| Angola | 36 | 36 | **100.0** |
| Zimbabwe | 72 | 72 | **100.0** |
| Other country | 6 | 4 | **66.7** |
| **Total** | **10,622** | **5,466** | **51.5** |
