# Supplementary material for: The illicit cigarette market in the Democratic Republic of the Congo (DRC): Findings from a cross-sectional study of empty cigarette packs
Source: PLOS Glob Public Health. 2025 Jun 25;5(6):e0003937. doi: 10.1371/journal.pgph.0003937 (PMC12194187; doi:10.1371/journal.pgph.0003937)
Supplement: S4 Text — (DOCX) [file pgph.0003937.s006.docx]

**S4 Table. Proportion of illicit empty packs by type of collection point and illicit criteria with all imported yellow stamps as illicit**

|  | **Type of collection point** | | | | | | | |
| --- | --- | --- | --- | --- | --- | --- | --- | --- |
|  | **Stationary retailer** | | **Mobile retailer** | | **Garbage bins/streets** | | **Total** | |
| **Criteria** | Illicit n (%) | Total | Illicit n (%) | Total | Illicit n (%) | Total | Illicit n (%) | Total |
| Tax stamp requirements | 3,542 | 7,116 | 823 | 1,522 | 1,101 | 1,984 | 5,466 | 10,622 |
|  | (49.8) |  | (54.1) |  | (55.5) |  | (51.5) |  |
| Written health warning | 656 | 7,116 | 29 | 1,522 | 163 | 1,984 | 848 | 10,622 |
|  | (9.2) |  | (1.9) |  | (8.2) |  | (8.0) |  |
| Notice of prohibition of sale to and by minors | 505 | 7,116 | 6 | 1,522 | 87 | 1,984 | 598 | 10,622 |
|  | (7.1) |  | (0.4) |  | (4.4) |  | (5.6) |  |
| Tar and nicotine content | 435 | 7,116 | 0 | 1,522 | 47 | 1,984 | 482 | 10,622 |
|  | (6.1) |  | (//) |  | (2.4) |  | (4.5) |  |
